# Combined deletion and DNA methylation result in silencing of *FAM107A* gene in laryngeal tumors

**DOI:** 10.1038/s41598-017-05857-1

**Published:** 2017-07-14

**Authors:** Katarzyna Kiwerska, Marcin Szaumkessel, Julia Paczkowska, Magdalena Bodnar, Ewa Byzia, Ewelina Kowal, Magdalena Kostrzewska-Poczekaj, Joanna Janiszewska, Kinga Bednarek, Małgorzata Jarmuż-Szymczak, Ewelina Kalinowicz, Małgorzata Wierzbicka, Reidar Grenman, Krzysztof Szyfter, Andrzej Marszałek, Maciej Giefing

**Affiliations:** 1 Institute of Human Genetics, Polish Academy of Sciences, Strzeszyńska 32, 60-479 Poznań, Poland; 2 Department of Tumor Pathology, Greater Poland Cancer Center, Garbary 15, 61-866 Poznań, Poland; 3 Department of Clinical Pathomorphology, Collegium Medicum in Bydgoszcz, Nicolaus Copernicus University in Toruń, Curie-Sklodowskiej 9, 85-094 Bydgoszcz, Poland; 4 Department of Otolaryngology and Laryngeal Oncology, Poznan University of Medical Sciences, Przybyszewskiego 49, 60-355 Poznań, Poland; 5 Department of Hematology and Bone Marrow Transplantation, Poznan University of Medical Sciences, Szamarzewskiego 82/84, 60-569 Poznań, Poland; 6 Department of Otorhinolaryngology, Head and Neck Surgery and Department of Medical Biochemistry, Turku University Central Hospital and Turku University, PO Box 52 FI-20521, Turku, Finland; 7 Department of Audiology and Phoniatrics, Poznan University of Medical Sciences, Przybyszewskiego 49, 60-355 Poznań, Poland; 8 Oncologic Pathology and Prophylaxis Poznan University of Medical Sciences & Greater Poland Cancer Center, Garbary 15, 61-866 Poznań, Poland

## Abstract

Larynx squamous cell carcinoma (LSCC) is characterized by complex genotypes, with numerous abnormalities in various genes. Despite the progress in diagnosis and treatment of this disease, 5-year survival rates remain unsatisfactory. Therefore, the extended studies are conducted, with the aim to find genes, potentially implicated in this cancer. In this study, we focus on the *FAM107A* (3p14.3) gene, since we found its significantly reduced expression in LSCC by microarray profiling (Affymetrix U133 Plus 2.0 array). By RT-PCR we have confirmed complete *FAM107A* downregulation in laryngeal cancer cell lines (15/15) and primary tumors (21/21) and this finding was further supported by FAM107A protein immunohistochemistry (15/15). We further demonstrate that a combined two hit mechanism including loss of 3p and hypermethylation of *FAM107A* promoter region (in 9/15 cell lines (*p* < 0.0001) and in 15/21 primary tumors (*p* < 0.0001)) prevails in the gene transcriptional loss. As a proof of principle, we show that Decitabine - a hypomethylating agent – restores *FAM107A* expression (5 to 6 fold increase) in the UT-SCC-29 cell line, characterized by high DNA methylation. Therefore, we report the recurrent inactivation of *FAM107A* in LSCC, what may suggest that the gene is a promising tumor suppressor candidate involved in LSCC development.

## Introduction

Larynx squamous cell carcinoma (LSCC) belongs to the large group of head and neck squamous cell cancers (HNSCC) and is still among the most often diagnosed tumors worldwide. Each year almost 10 persons per 100 000 develop this disease^1^. Invariably, these tumors are commonly detected at an advanced stage, what results in poor prognosis, followed by adverse outcomes. Standard therapy usually includes surgical resection with or without post-operative radio- or chemotherapy. Such procedure, together with the poor targeted therapy limited only to the drugs against EGFR receptor, results in low five-year survival rate^1–3^. Due to the complex alterations acquired in multistep process of HNSCC carcinogenesis, the tumor itself is very heterogeneous and is characterized by a number of genetic changes (except for HPV-related tumorigenesis with minor genetic lesions)^4, 5^. The genetic and epigenetic changes interplay at different stages of carcinogenesis, leading to deregulation of key genes, like already known oncogenes: *EGFR*, *CCND1*, *MYC* and *PIK3CA* or tumor suppressor genes: *TP53*, *CDNK2A* and *NOTCH1* ^6–10^. However, finding novel, potential biomarkers may not only extend the knowledge about genetic background of laryngeal cancers, but also may help to estimate the risk of disease burden or the response to the applied therapy.

The application of novel high throughput technologies based on microarrays and next generation sequencing has accelerated tumor genetic studies in recent years^10, 11^. In this study we have used expression profiles from Affymetrix GeneChip Human Genome U133 Plus 2.0 arrays, performed previously by our group^12^. To identify novel, potential tumor suppressor genes, we screened this data for genes showing reduced or lack of the mRNA expression. This enabled us to indicate the *FAM107A* gene as a promising tumor suppressor gene candidate involved in larynx cancer development and to identify the main mechanisms of its inactivation in this type of cancer.

## Results

### The microarray based expression analysis revealed downregulation of *FAM107A* in laryngeal tumor cell lines and primary samples

**Figure 1:**
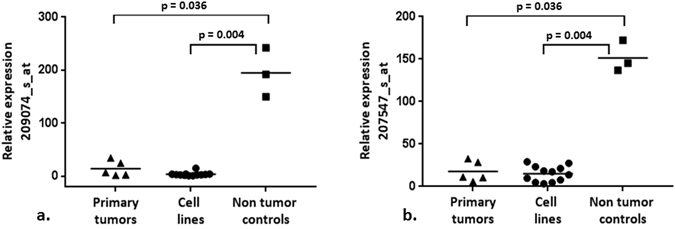
The relative expression of *FAM107A* indicated by two tags – (**a**) 209074_s_at and (**b**) 207547_s_at. Significantly downregulated expression of *FAM107A* in laryngeal primary tumors and cell lines is shown (p < 0.05; U Mann Whitney test; GraphPad Prism 7 demo version).

Using the expression profiles established previously with the application of Affymetrix GeneChip Human Genome U133 Plus 2.0 array we have searched for genes differentially expressed in all laryngeal cancer samples (12 cell lines and 5 primary tumors; n = 17) in comparison to 3 non-tumor controls. For this purpose, we have screened the microarray data for tags carrying the “absent” call in each laryngeal cancer sample and the “present” call in each non-tumor control. This filtering resulted in 11 out of 54 675 tags that fulfilled these criteria and included 209074_s_at, that corresponded to *FAM107A* gene localized in 3p14.3 chromosomal region (chr3:58,549,845–58,563,491; UCSC Genome Browser GRCh37/hg19). This gene was selected for further study because in its case, the difference in mean expression level between tumor and non-tumor samples was the highest. Another tag, namely 207547_s_at also corresponded to this gene. Figure 1 presents the relative expression of *FAM107A* as indicated by both tags in the analyzed samples, showing their statistically significant downregulation in the laryngeal cancer samples (both primary tumors and cell lines) in comparison to non-tumor controls (Mann-Whitney test). Using the 209074_s_at tag we have identified a 48.6 fold (p = 0.004) and 13.5 fold (p = 0.036) decrease in expression in cell lines and primary tumor samples, respectively as compared to non-tumor controls. Likewise, 10 and 8.5 fold decrease of expression was observed for 207547_s_at tag (p = 0.004 and p = 0.036, respectively). Intrigued by this finding, we have further evaluated the status of *FAM107A* copy number on Agilent Human Genome CGH 244A and 44K Microarrays (aCGH), performed previously on 13 cell lines. Homozygous deletions targeting this gene were excluded earlier^12, 13^, however basing on the copy number plots presenting chromosome 3 in 13 cell lines we have observed deletions of the short arm of this chromosome, resulting in loss of one copy of the *FAM107A* gene (Supplementary Figure S1).

### RT-PCR confirms the complete loss of *FAM107A* expression in laryngeal cancer samples

**Figure 2:**
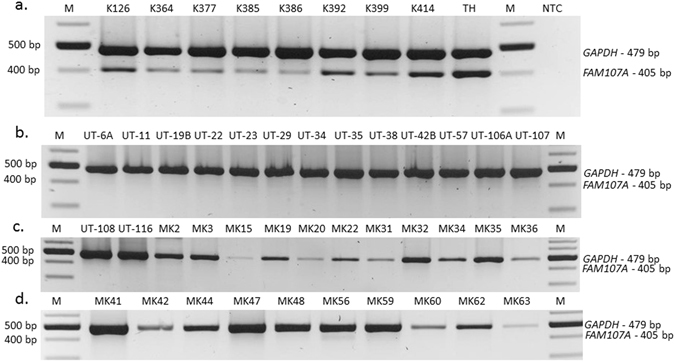
Retained expression of *FAM107A* in non-tumor controls (K samples; panel a) and lack of *FAM107A* expression in laryngeal cancer samples: cell lines (UT samples; panel b and c) and primary tumors (MK samples; panel c and d). *GAPDH* expression was used as the internal control. NTC – no template control. Size marker Perfect^TM^ 100–1000 bp DNA Ladder (Eurx).

To confirm the downregulation of *FAM107A* indicated by the expression microarray we have amplified the whole coding region of this gene by RT-PCR in the presence of *GAPDH* gene as the internal control. Because the expression microarray was performed on both, cell lines and primary tumors we found it to be reasonable to include in this experiment both types of samples. The results, confirmed the complete lack of *FAM107A* expression in laryngeal cancer cell lines (15/15; 100%; Fig. 2b,c) and primary tumors (21/21; 100%; Fig. 2c,d). On the contrary, two bands, specific for both, *FAM107A* and *GAPDH* genes were visible in all applied non-tumor controls (9/9; Fig. 2a). This finding encouraged us to search for the potential mechanism inactivating the remaining copy of *FAM107A*.

### Sequencing of coding exons and intron/exon junctions of *FAM107A*

To find the potential loss of function mutations in *FAM107A* we have performed Sanger sequencing in 15 LSCC cell lines. The coding sequences of *FAM107A* (NM_001076778.2), covering exon 2, 3 and 4, together with 5′ and 3′ splicing sites (up to 8 intronic bp) were analyzed. Four different single nucleotide polymorphisms (SNP) were detected in four different cell lines (Table 1 and Supplementary Figure S2). All identified variants were homozygous, further corroborating the deletion of one copy observed by aCGH. Three of them were missense variants (rs1043942, rs11539086 and rs141609445) – resulted in amino acid change and one was synonymous change (rs1139701). The rs1043942 and rs141609445 missense variants were indicated during *in silico* analysis with the application of PolyPhen-2 software as probably damaging for the protein structure. Additionally, the analysis of population data for the latter revealed, that in European-American population only the reference allele was observed (http://browser.1000genomes.org).

**Table 1:** The molecular variants detected in cell lines and their potential effect on the *FAM107A* gene (1000 Genomes database).

Molecular variant	Cell line	Sequence alteration (NM_001076778.2)	Protein change (NP_001070246.1)	Localization (GRCh37/hg19)	Type of mutation	PolyPhen-2	Allele frequency [EUR]
rs1043942	UT-SCC-116	c.265 G > T	p.Ala89Ser	chr3:58,552,997	missense	probably damaging	5%
rs1139701	UT-SCC-19B	c.312 G > A	p.Gln104	chr3:58,552,950	synonymous	—	5%
rs11539086	UT-SCC-23	c.421 G > C	p.Glu141Gln	chr3:58,552,329	missense	benign	7%
rs141609445	UT-SCC-42B	c.395 G > A	p.Arg132Gln	chr3:58,552,355	missense	probably damaging	0%

While no clear novel inactivating mutations were detected in this analysis, we have discontinued searching for molecular variants in primary laryngeal tumor samples.

### DNA methylation analysis in the *FAM107A* promoter region by bisulfite pyrosequencing

**Figure 3:**
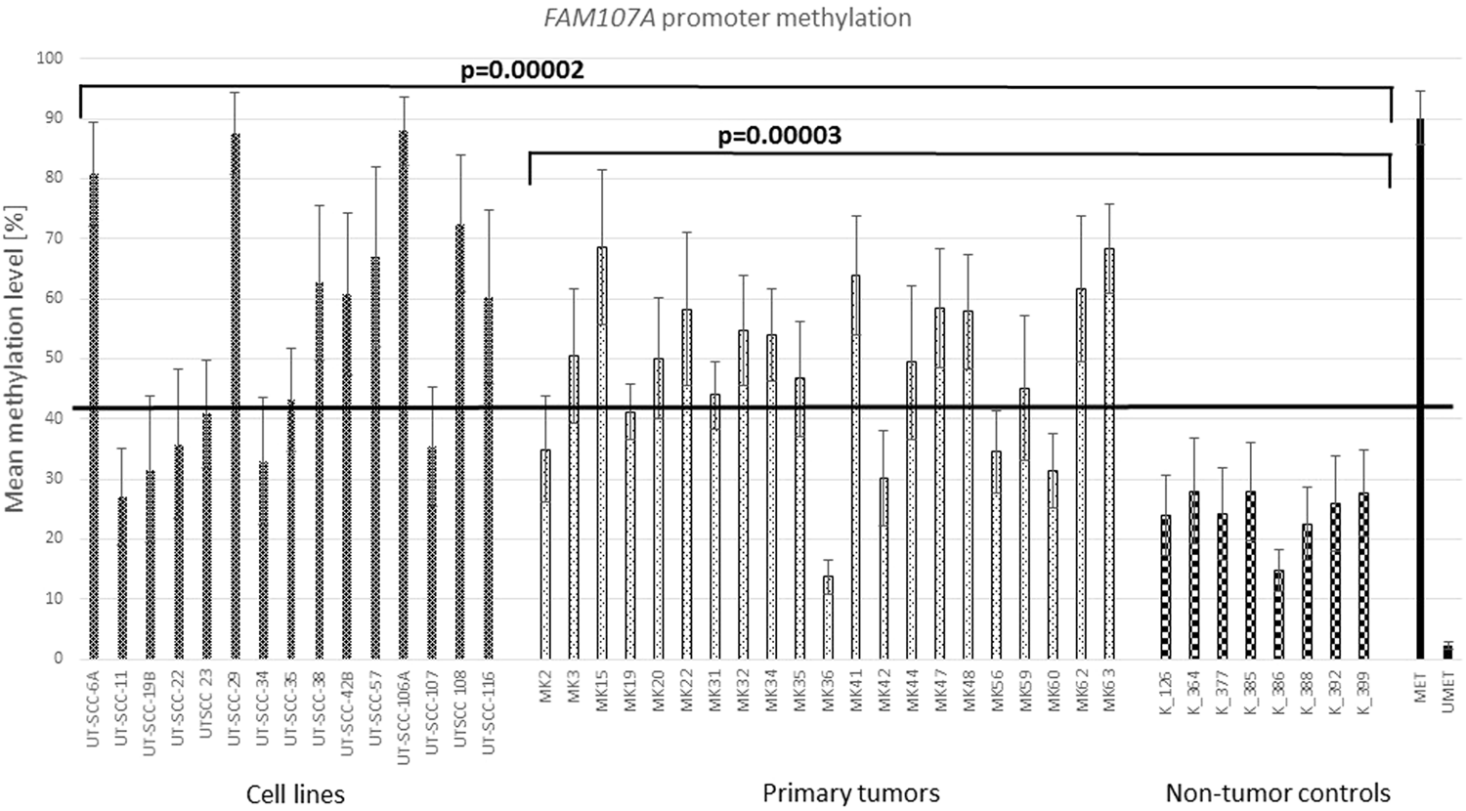
Mean methylation of *FAM107A* promoter region in analyzed cell lines, primary tumors and non-cancer controls. The flat black line indicates the cut off value of 41%, above which samples are assigned as hypermethylated.

To study further the potential mechanisms underlying *FAM107A* downregulation we have analyzed the DNA methylation level of this gene in 15 cell lines, 21 primary tumors and 8 non-tumor controls (Fig. 3). The data obtained for non-tumor controls were used to calculate the cut-off value (41%), above which the samples were assigned as hypermethylated. The dilution series of methylated and unmethylated templates were used to determine the assay sensitivity and PCR bias. It showed lowered selectivity of the primers towards methylated template and thus the cut-off point assigned at 41% of methylation corresponded to the mix of standards containing 70% of methylated template. Therefore, all methylation results above the cut-off point were considered as hypermethylated (Supplementary Figure S3).

The methylation level ranged from 15 to 28% for non-tumor controls, 27% to 87% for cell lines and 14% to 69% for primary tumors. In detail, the pyrosequencing analysis revealed significant differences in DNA methylation level between tumor samples (both laryngeal cancer cell lines and primary tumors) and non-tumor controls (Fig. 3). The hypermethylation was detected in 9/15 cell lines (60%; p < 0.0001) and in 15/21 primary tumors (71%; p < 0.0001).

### Demethylation of UT-SCC-29 cell line with Decitabine

**Figure 4:**
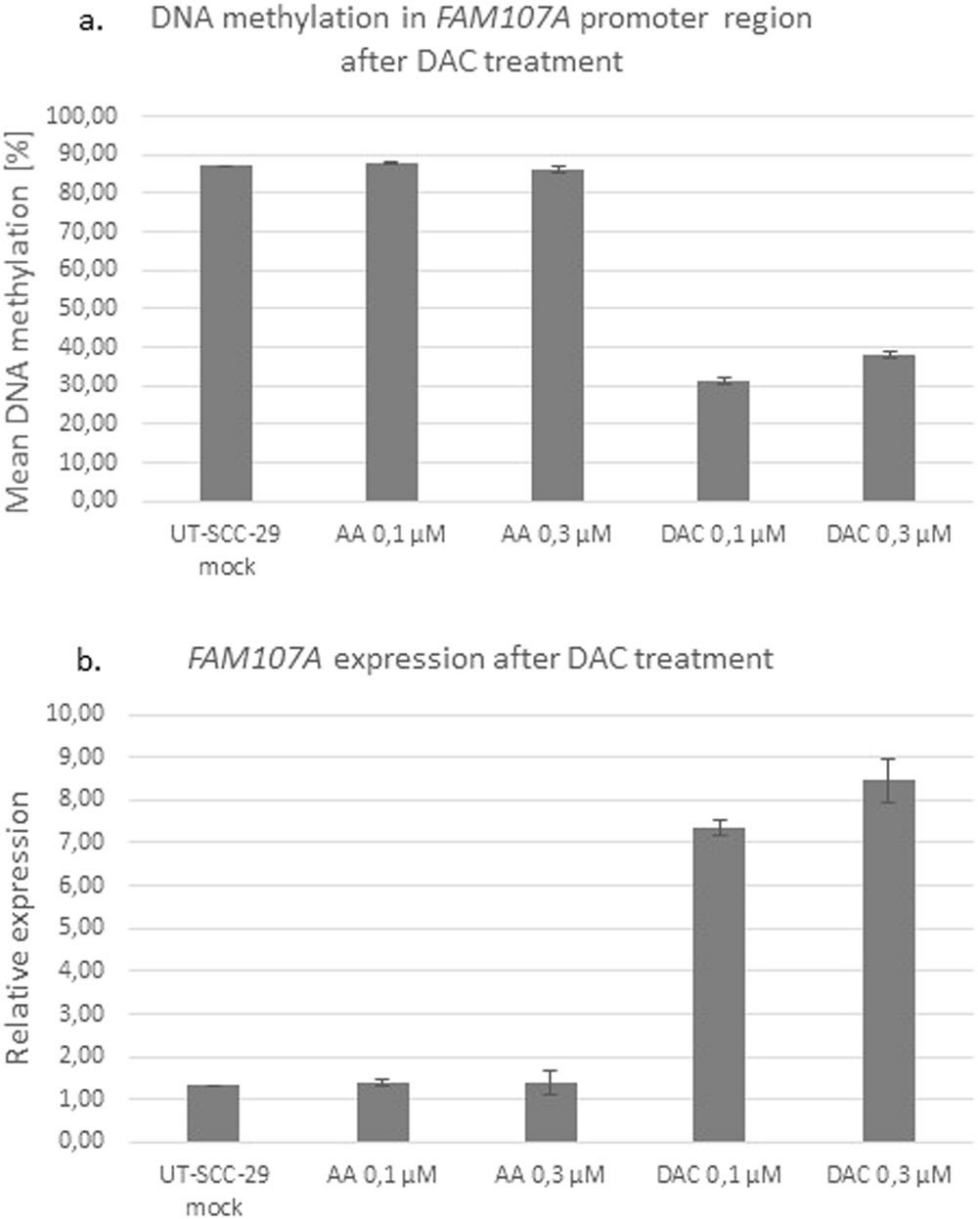
*FAM107A* DNA methylation (**a**) and mRNA expression (**b**) after DAC treatment (demethylation).

The pyrosequencing analysis revealed that UT-SCC-29 cell line showed the highest DNA methylation level for *FAM107A* in our study group. Therefore, we decided to demethylate this cell line with the use of Decitabine (DAC; 5-aza-2′-deoxycytidine; Sigma-Aldrich) - a known hypomethylating agent. Further, we have analyzed DNA methylation level in the promoter region of *FAM107A* gene, revealing its significant reduction in samples treated with DAC (Fig. 4a). Differences between mean methylation (MM) values of untreated UT-SCC-29 cell line (MM = 87%) and those treated with 0.1 μM DAC (MM = 31%) and 0.3 μM DAC (MM = 38%) were 56% and 49%, respectively. On the contrary, there was no noticeable difference between mean methylation (MM) values of untreated UT-SCC-29 cell line (MM = 87%) and those treated with 0.1 μM and 0.3 μM acetic acid (MM = 88% and 86%, respectively) – a solvent of DAC, having no influence on methylation or demethylation process (Fig. 4a).

Next, cDNA from the same set of samples was applied for RT-qPCR to analyze *FAM107A* expression after the demethylation. The obtained data revealed, that in UT-SCC-29 cell line treated with either 0.1 μM DAC or 0.3 μM DAC, *FAM107A* expression is restored (Fig. 4b). The relative expression values increased 5 to 6 fold in comparison to UT-SCC-29 samples untreated (“mock”) or to treated with acetic acid, where the expression is barely observed (Fig. 4b), suggesting an important role of *FAM107A* epigenetic silencing in LSCC.

### Evaluation of FAM107A protein expression by immunohistochemistry

**Figure 5:**
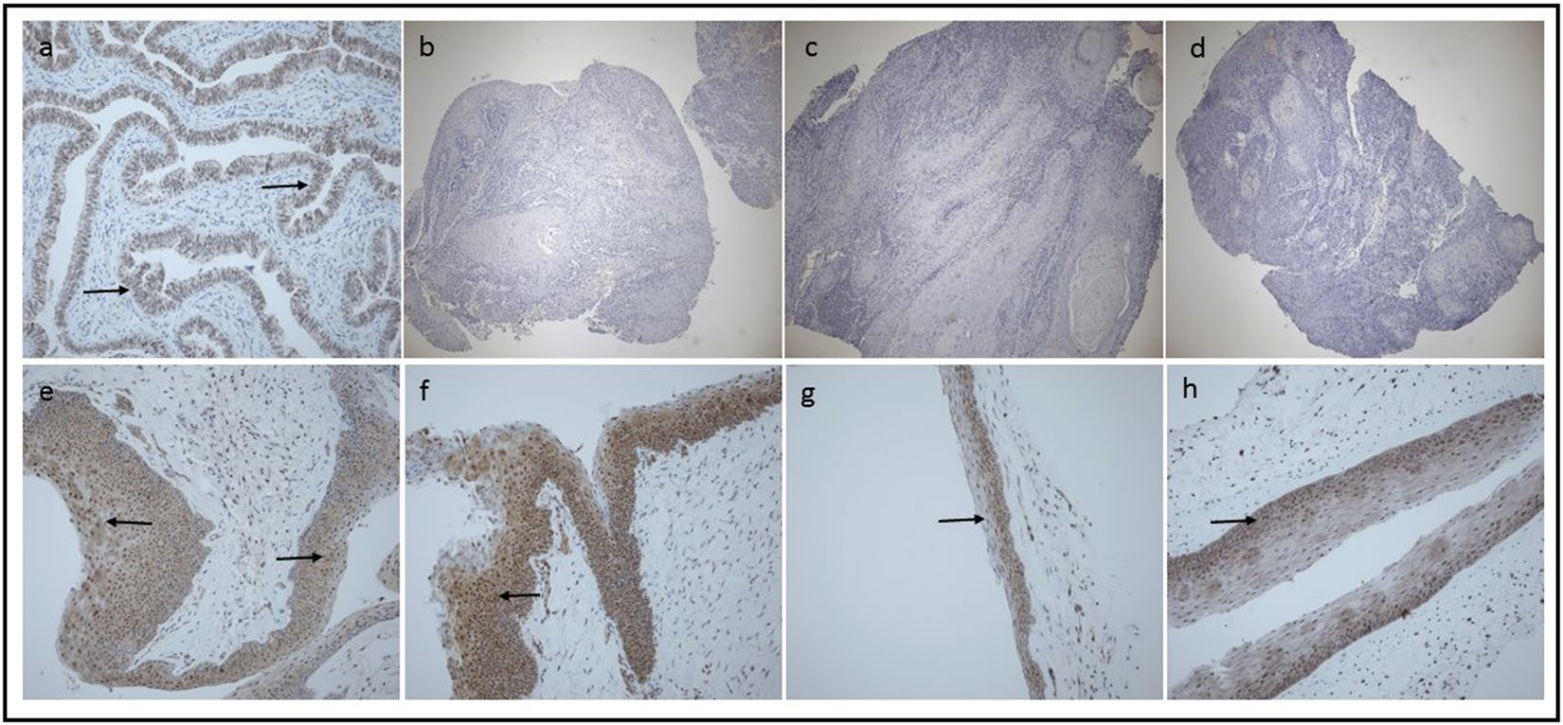
Microphotograph of immunohistochemically stained FAM107A protein in: fallopian tube (control) (**a**), LSCC (**b**–**d**) and non-tumor controls (**e**–**h**). Nucleus counterstained with hematoxylin, arrows indicate positive nuclear/cytoplasmic staining (brown), primary objective magnification 10x.

The nuclear/cytoplasmic expression of FAM107A protein was found in glandular cells of fallopian tube (Fig. 5a), which served as the positive control for antibody binding. The FAM107A immunostaining revealed lack of the protein expression in all analyzed primary LSCC samples (15/15; 100%; Fig. 5b–d), while in all non-tumor controls (5/5; 100%; Fig. 5e–h) nuclear/cytoplasmic FAM107A protein expression was found.

### The relationship between DNA methylation of *FAM107A* and tumor stage

We have assessed the relationship between DNA methylation of *FAM107A* and: tumor size (T), nodal involvement (N) and differentiation degree (G), however, there was no significant association between any of the analyzed parameters (Table 2). We were not able to analyze the impact of *FAM107A* hypermethylation on patients’ survival due to the lack of relevant data.

**Table 2:** The relationship between the *FAM107A* methylation and tumor stage.

Variable	Determinant	Material	Hypermethylation		Significance (*p* value)
			Positive	Negative	
T stage	T1 vs T2 + T3 + T4	Cell lines	n (T1) = 1 n (T2 + T3 + T4) = 8	n (T1) = 2 n (T2 + T3 + T4) = 4	0,69
		Primary tumors	n (T1) = 0 n (T2 + T3 + T4) = 15	n (T1) = 0 n (T2 + T3 + T4) = 6	0,81
		Cell lines + primary tumors	n (T1) = 1 n (T2 + T3 + T4) = 23	n (T1) = 2 n (T2 + T3 + T4) = 10	0,52
N stage	N0 vs N1 + N2 + N3	Cell lines	n (N0) = 6 n (N1 + N2 + N3) = 3	n (N0) = 5 n (N1 + N2 + N3) = 1	0,91
		Primary tumors	n (N0) = 6 n (N1 + N2 + N3) = 9	n (N0) = 1 n (N1 + N2 + N3) = 5	0,61
		Cell lines+ primary tumors	n (N0) = 12 n (N1 + N2 + N3) = 12	n (N0) = 6 n (N1 + N2 + N3) = 6	0,72
Degree of differentiation	G1 + G2 vs G3	Cell lines	n (G1 + G2) = 7 n (G3) = 2	n (G1 + G2) = 6 n (G3) = 0	0,64
		Primary tumors	n (G1 + G2) = 12 n (G3) = 3	n (G1 + G2) = 6 n (G3) = 0	0,62
		Cell lines + primary tumors	n (G1 + G2) = 19 n (G3) = 5	n (G1 + G2) = 12 n (G3) = 0	0,23

## Discussion

The solid tumor formation is complex and associated with acquisition of multiple genetic alterations, which influence and deregulate the different cellular processes^14^. Within this, tumor suppressor genes (TSGs) are frequently inactivated at the early stages of disease development. Their loss of anticancer functions is preceded by two events leading to inactivation of each allele, as described by Knudson^15^.

20 years ago Califano *et al*. have shown that one of the earliest steps of head and neck tumor development, namely the transition from hyperplastic mucosa to dysplasia is related to the loss of heterozygosity (LOH) at 3p chromosomal arm^4^. In line with the observation by Califano et. al, several genes localized in 3p arm, known to be engaged in head and neck cancer appearance, e.g. *FHIT* or *RASSF1A* have been identified^16–18^.

In this study we have focused on a novel TSG candidate localized in 3p14.3 region, named *FAM107A* (*Homo sapiens* family with sequence similarity 107, member A; aliases *TU3A* or *DRR1*), potentially involved in laryngeal carcinogenesis. We have shown recurrent transcriptional loss of *FAM107A* in LSCC using microarray data and RT-PCR (Figs 1 and 2). This is in concordance with the results obtained by other authors, who showed that *FAM107A* (previously known as *TU3A* or *DRR1*) is downregulated in various tumors and cell lines^19–22^. However, we are the first who show that *FAM107A* is also downregulated in laryngeal tumors. Moving forward, we have evaluated the DNA copy number of *FAM107A* using the array CGH data generated previously^12, 13^. Although the homozygous deletions including this gene were previously excluded^12^, it is well known, that LOH affecting 3p chromosomal arm is a frequent event in the early step of HNSCC development^4, 16, 18, 23^. The analysis of copy number profiles of chromosome 3 in the analyzed cell lines supports this assumption (Supplementary Figure S1).

Given the above-mentioned arrangements we were interested in finding the potential mechanism underlying *FAM107A* inactivation in laryngeal tumors, considering that other mechanisms, like point mutations or DNA methylation may affect the remaining gene copy. At first we have investigated the mutational status of this gene in 15 laryngeal cancer cell lines. We found four different sequence variants in four different cell lines – three missense and one synonymous (Table 1 and Supplementary Figure S2). One of them, namely rs141609445 resulting in replacing Arginine by Glutamine is poorly analyzed and the 1000 Genomes database indicates that only the reference allele is observed in European-American population. Moreover, PolyPhen-2 online predicting tool shows that this sequence variant is probably damaging for the structure and function of encoded protein. Nevertheless, the sequencing result suggest that *FAM107A* is not necessarily inactivated by mutations, what is in line with other data, including cBioPortal for Cancer Genomics^19, 24, 25^.

As the mutation analyses did not reveal a potential mechanism behind *FAM107A* silencing, in the next step we have analyzed the methylation level of *FAM107A* promoter region. With the application of bisulfite pyrosequencing we have shown that 60% of cell lines (9/15) and 71% of primary tumors (15/21) are hypermethylated and that the differences in mean methylation value between analyzed samples and non-tumor controls are statistically significant (p < 0.001; Fig. 3). The hypermethylation was detected among others in cell lines UT-SCC 42B and UT-SCC 116, in which, by Sanger sequencing we have revealed two, probably damaging sequence variants (Table 1). On the other hand, cell lines UT-SCC 19B and UT-SCC 23, harboring synonymous and missense (benign by PolyPhen-2) variants respectively, were not hypermethylated (Table 1 and Fig. 3). Both findings further supports that *FAM107A* is not inactivated by mutation or SNP. The high prevalence of recurrent *FAM107A* hypermethylation in the analyzed samples indicates with high degree of certainty that it is the predominant mechanism responsible for the transcriptional silencing of the remaining copy of the gene. Nevertheless, the available data concerning *FAM107A* methylation in tumors are inconsistent. Awakura *et al*. showed that more than 40% of renal cell carcinomas presents methylated *FAM107A* promoter^21^. This gene was also indicated as hypermethylated in hepatocellular carcinoma^26^. On the contrary, in the group of 60 lung cancer patients with decreased *FAM107A* expression, DNA methylation was observed in minor amount of samples and thus was excluded by the authors as the mechanism of its inactivation^27^. However, in the latter case, the use of the less sensitive MSP method (methylation-specific PCR) to primary tumors might introduce the bias in detecting methylation.

Intrigued by the fact, that the UT-SCC-29 cell line was almost completely methylated in our study (Fig. 3), we took an attempt to demethylate this cell line using well-known hypomethylating agent: 5-aza-2′-deoxycytidine (DAC) and subsequently to verify *FAM107A* methylation and expression. The used reagent – Decitabine – is currently applied in the epigenetic therapy of various hematologic malignancies. It acts through the whole genome (not selectively) as an inhibitor of DNA methyltransferase 1 (DNMT1), leading to the reactivation of silenced genes^28^. In our study, treatment with DAC resulted in significant decrease of *FAM107A* methylation, while it was preserved at the high level in control samples, untreated with DAC (Fig. 4a). This shows, that *FAM107A* methylation is reversible. Moreover, the subsequent RT-qPCR revealed that *FAM107A* expression was restored in samples treated with DAC (Fig. 4b). This result confirms that DNA methylation is responsible for *FAM107A* transcriptional inactivation and raises direct question whether DNA methylation is associated with cancer stage. In the study of Awakura *et al*. the authors found that DNA hypermethylation of *TU3A* (former name of *FAM107A*) corresponded with tumor stage (>T2) in primary renal cell carcinoma, however they did not observe such relationship for primary bladder and testicular cancers^21^. Moreover, the tumor differentiation did not correlate with DNA methylation status in any of the analyzed tumors. Our study confirmed lack of relationship between tumor extension, nodal involvement and tumor differentiation with *FAM107A* hypermethylation (Table 2). But it should be emphasized, that in our study group, the primary samples originated predominantly from patients with advanced tumor stage, thus if *FAM107A* silencing took place at early stage of carcinogenesis, we were not able to observe such association. Thus, it might be fruitful to assess *FAM107A* expression or methylation status in premalignant lesions of the larynx.

The shown recurrent inactivation of the gene in LSCC is especially interesting in relation to the potential function of *FAM107A*. Although it is not completely understood so far, Wang *et al*. have shown that the protein encoded by the gene contains a nuclear localization signal and a coiled domain, suggesting that it may play a role in regulation of gene transcription and signal transduction^19^. In our study we confirmed the nuclear and cytoplasmic expression of this protein in non-tumor control mucosa (Fig. 5e–h) and importantly showed the lack of FAM107A expression in laryngeal tumor samples (Fig. 5b–d). Functional studies of Wang *et al*. and Liu *et al*. revealed that the re-expression of *DRR1* gene (former name of *FAM107A*) in renal and lung cancer cell line respectively, resulted in growth suppression and apoptosis^19, 29^. The same effect, i.e. tumor growth inhibition and elevation of apoptosis was observed in the xenograft model after injection of nude mice bearing A549 tumor cells with vector containing *DRR1* cDNA^29^.

In summary, our study indicates, that in the course of laryngeal squamous cell carcinoma, *FAM107A* is inactivated by combined deletions and DNA methylation events. Considering its downregulation in multiple tumors we postulate, that its inactivation is not specific to a given cancer (like laryngeal cancer) but is in contrast, a widespread phenomenon.

## Material and Methods

### Cell lines

Altogether, 17 laryngeal cancer cell lines derived from patients treated at Turku University Central Hospital (Finland) were included in this study. The cell lines were previously cytogenetically characterized, among others by Jarmuż *et al*. and Jarvinen *et al*.^30, 31^. Cells were grown in 25 cm^2^ flasks in Dulbecco’s modified Eagle’s medium supplemented with 10% of fetal bovine serum at 37 °C under 5% CO_2_ atmosphere. For DNA and RNA isolation cell lines were cultured to reach 80% of confluence and subsequently harvested with 0.1% trypsin and 0.2% EDTA. The detailed characteristic of cell lines is presented in Supplementary Table S1.

### Primary tumors

The group of 21 primary laryngeal tumor samples derived from patients who underwent surgery in the Department of Otolaryngology and Laryngological Oncology, Poznan University of Medical Sciences was used in this study. All samples collected during the surgery were cut into three parts:

(I) for histopathological assessment and IHC analysis (into the probe filled with 10% of buffered formalin),
(II) for DNA isolation (required immediate freezing at −80 °C in an empty tube) and
(III) for RNA analysis (stored in RNA*later* solution (Sigma-Aldrich, Saint Louis, USA) until the isolation step).

Samples containing more than 60% of tumor cells were included into the study. All experiments were performed in accordance with relevant guidelines and regulations approved by the Ethics Review Board of Poznan University of Medical Sciences (decision no. 164/10 and 502/15) and informed consent was obtained from all donors. The clinical data of patient and tumor characteristics are presented in Supplementary Table S2.

### Non-tumor controls

Depending on the performed analysis, various non-tumor samples were applied as controls in this study:

(I) for microarray expression analysis three non-tumor controls were applied, i.e. human larynx total RNA (Stratagene, Agilent Technologies, Santa Clara, USA) and RNA derived from bronchial airway epithelia reconstituted *in vitro* (two donors) (EC, Epithelix Sarl, Geneve, Switzerland) – both commercially available, as well as LX10 – histologically normal mucosa from surgical margin,
(II) for RT-PCR analysis nine non-tumor controls were applied, i.e. eight samples obtained during the uvulopalatoplasty procedure (nonmalignant ailments connected with snoring) as well as commercially available total RNA collected from different adult human tissues (qPCR Human Reference Total RNA, Clontech Laboratories, Mountain View, USA), thus providing the broadest coverage of the expressed genes,
(III) for bisulfite pyrosequencing analysis eight non-tumor controls collected during the uvulopalatoplasty procedure were applied. Aditionally, the fully methylated standard (MET, Millipore, Hilden, Germany) and unmethylated DNA (UMET), i.e. the whole genome amplified DNA from pooled peripheral blood lymphocytes were used in each run,
(IV) for immunohistochemistry (IHC) five samples of Reinke’s edema (nonmalignant ailments of the larynx) and one sample of fallopian tube were applied.

### DNA and RNA isolation

The nucleic acids from all cell lines, primary tumor samples and non-tumor controls were isolated with the application of standard methods: DNA was obtained using phenol/chloroform extraction and ethanol precipitation and RNA was isolated according to Chomczynski’s method with application of Trizol reagent^32^. The whole genome amplified DNA from pooled peripheral blood lymphocytes was prepared with GenomePlex® Whole Genome Amplification Kit (Sigma-Aldrich), according to manufacturer’s procedure. The concentration and purity of nucleic acids were assessed using NanoDrop-1000 spectrophotometer and RNA integrity was estimated with the use of RNA 6000 Nano Kit on Agilent 2100 BioAnalyzer (Agilent). DNA was stored at −20 °C and RNA at −80 °C until the laboratory use.

### Microarray-based gene expression and copy number analysis

The microarray-based analyses were performed by our group previously and the detailed description of the respective procedures is published elsewhere^12, 13^. The expression analysis was performed using Affymetrix GeneChip Human Genome U133 Plus 2.0 array and the genome-wide analysis of DNA copy number changes was performed with the use of Agilent Human Genome CGH 244 A and 44 K Microarray Kits (Agilent Technologies, Waldbronn, Germany).

### Reverse transcription and PCR

1 µg of total RNA from 15 cell lines, 21 primary tumors and 9 non-tumor controls was reverse transcribed to cDNA with the application of Maxima First Strand cDNA Synthesis Kit (Thermo Fisher Scientific, Waltham, Massachusetts, USA), according to the manufacturer’s procedure. In the next step, the level of *FAM107A* expression was analyzed by multiplex RT-PCR. Two primer pairs were designed: one, covering the whole coding sequence of *FAM107A* gene and second, covering exons 5–8 of *GAPDH* gene (Table 3). *GAPDH* fragment constituted an internal control of RNA integrity and appropriate PCR reaction. In both cases, the primers were designed with the use of Primer3 (v. 0.4.0) online tool (http://bioinfo.ut.ee/primer3-0.4.0/) in a way to encompass all transcript variants indicated in UCSC Genome Browser (hg19; http://genome-euro.ucsc.edu/). The primer sequences were verified with primer BLAST database (http://blast.ncbi.nlm.nih.gov/Blast.cgi) to confirm their specificity. Total volume of 10 µl multiplex PCR mixture consisted of: 1x *Taq* Buffer with (NH_4_)_2_SO_4_, 0.2 mM of each dNTP, 20 pmol of each *FAM107A* primer, 5 pmol of each *GAPDH* primer, 2 mM of MgCl_2_, 1.25U of *Taq* DNA Polymerase (recombinant) and 0.5 µl of cDNA. Primers were synthesized in Genomed S.A. Company (Warsaw, Poland) and the other reagents were purchased from Thermo Fisher Scientific. The reactions were performed in DNA Engine DYAD Peltier Thermal Cycler (BIO-RAD, Hercules, USA) and the reaction conditions are presented in Supplementary Table S3. Afterwards, 5 µl of each PCR product was mixed with 6x Loading Dye (Thermo Fisher Scientific) and run on 2% agarose gel stained with ethidium bromide (1 h and 40 min, 70 V) in the presence of Perfect 100–1000 bp DNA Ladder (EURx, Gdansk, Poland). The results were visualized under UV light (BioDoc-it Imaging System, UVR, USA). Two bands on the gel were expected in samples showing *FAM107A* expression: 479 bp for *GAPDH* gene and 405 bp for *FAM107A* gene, while only the longer one was expected in samples lacking *FAM107A* expression.

**Table 3:** Primer sequences, genomic localization of the amplified sequences (*indicates genomic position including introns) and reaction conditions.

Analysis	Gene	Position (GRCh37/hg19)	Primer sequence	Product length	Tm
RT-PCR	FAM107A	*chr3:58,552,316–58,555,558	F: AGACATTGGGGGCCTGAT	405 bp	55 °C
			R: TACAGCTCTCTCTCTTCGCTGGT		
	GAPDH	*chr12:6,646,327–6,647,090	F: ATGTTCGTCATGGGTGTGAA	479 bp	
			R: TCGCTGTTGAAGTCAGAGGA		
Sanger sequencing (NM_001076778)	FAM107A_ex2	chr3:58,555,318–58,555,696	F: CCAAGGTCCATCTGACATGA	379 bp	65 °C
			R: CCAGGATGAAAGCCAGCTC		
	FAM107A_ex3	chr3:58,552,824–58,553,187	F: AATAATGGGGGTTGGTGTCA	364 bp	
			R: TGCTCTGTCTGCTGATCCTC		
	FAM107A_ex4	chr3:58,552,192–58,552,548	F:CAAAACTCATCCCCAGGTTG	357 bp	
			R: ACAGAAGCAGGTGGGAACAT		
Bisulphite pyrosequencing	FAM107A	chr3:58563429–58563867	F: AAGGGAGGGGAAATTGTT	119 bp	55 °C
			R: Biotin-TAGTATTGGGTTAGTTTTTAA		
			S: ACTCCTCTATAAAATTCCAATAACAT		
RT-qPCR	FAM107A	*chr3:58,552,363–58,553,024	F: ATCAAGAAGAAGAAGGAG	150 bp	52 °C
			R: TTCCCTGACTTTAATAAAC		
	GAPDH	*chr12:6,645,659–6,645,955	F: GTCGGAGTCAACGGATT	220 bp	55 °C
			R: CCTGGAAGATGGTGATGG		

### Sequencing analysis

To perform the mutational analysis, three primer pairs covering three coding exons, including intron-exon junctions of *FAM107A* gene were designed using Primer3 (v. 0.4.0) online tool. The sequences of all primers are listed in Table 3. The PCR reaction was carried out in a total volume of 10 µl containing: 1x *Taq* Buffer with (NH_4_)_2_SO_4_, 0.2 mM of each dNTP, 10 pmol of each primer, 1.5 mM of MgCl_2_, 1.25U of *Taq* DNA Polymerase (recombinant) and 25 ng of DNA. Primers were synthesized in Genomed S.A. Company and all other consumables were purchased from Thermo Fisher Scientific. The reaction conditions are presented in Supplementary Table S3. All reactions were performed in DNA Engine DYAD Peltier Thermal Cycler (BIO-RAD). PCR products were verified during electrophoresis and purified with the use of 10 U of Exonuclease I and 1 U of FastAP^TM^ Thermosensitive Alkaline Phosphatase (both Thermo Fisher Scientific) and subsequently sequenced with the use of Big Dye Terminator Sequencing Kit Cycle v3.1 (Applied Biosystems, Inc. (ABI), Foster City, CA, USA), according to the manufacturer’s protocol. Hereafter, the PCR sequencing products were precipitated with ethanol, dissolved in 12 µl of Hi-Di Formamide and separated using ABI PRISM 310 Genetic Analyzer (Applied Biosystems). The results were analyzed using Sequencing Analysis v. 5.4 software and the CodonCode Aligner software (demo mode) in the presence of the reference sequence of *FAM107A* gene (NM_001076778.2 from RefSeq database; UCSC Genome Browser GRCh37/hg19).

### DNA methylation analysis by bisulfite pyrosequencing

**Figure 6:**
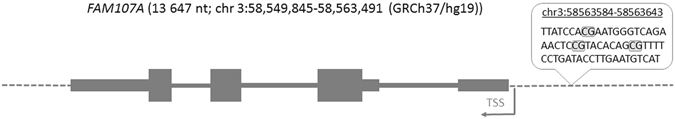
The structure of *FAM107A* gene. Three marked CG dinucleotides within the promoter region of the gene were amplified for DNA methylation (intron - exon not to scale). TSS –Transcription Start Site.

500 ng of purified DNA from 15 cell lines, 21 primary tumors and 8 non-tumor controls was converted with bisulfite solution using EZ DNA Methylation – Gold^TM^ Kit (Zymo Reasearch, Germany), according to the manufacturer’s protocol. The primers for pyrosequencing assay were designed with PyroMark Assay Design Software 2.0.1.15 (Qiagen). The reverse primer was 5′-biotinylated (Table 3). PCR was performed using PyroMark PCR kit (Qiagen) and the reaction mixture (25 µl) composed of: 1x PyroMark Master Mix (contains HotStarTaq DNA Polymerase, 1x PyroMark PCR Buffer and dNTPs), 10 pmol of each primer, 1x CoralLoad Concentrate and 1 µl of converted DNA. The reaction conditions are presented in Supplementary Table S3. PCR products were run on 2% agarose gel and were visualized under UV light. Pyrosequencing was performed with the use of PyroMark Q24 sequencer (Qiagen), including the fully methylated (MET) and unmethylated (UMET) controls. Three CG dinucletides (CG1 - chr3: 58,563,591–592; CG2 - chr3: 58,563,609–610 and CG3 - chr3: 58,563,618–619; Fig. 6), localized in the promoter region of *FAM107A* gene were analyzed for methylation level. The detailed sequencing protocol was described elsewhere^33^. Mean methylation level was calculated from the analyzed CG dinucleotides and was treated as *FAM107A* methylation in a given sample. Due to the technical impediments we quantified mean methylation from only two CG nucleotide repeats in the primary tumor samples, while all three CG repeats were analyzed in the remaining samples.

To evaluate which samples are hypermethylated the cut off value was determined, based on the results collected for non-tumor controls (three times the standard deviation of methylation for control samples + the highest value of DNA methylation observed in controls). Additionally, dilution series of commercially available methylated DNA template in unmethylated DNA template (WGA - from 0% to 100%, every 10%; Supplementary Figure S3) were used to estimate the extent of PCR bias and the sensitivity of the assay to measure DNA methylation levels in the analyzed samples.

### Decitabine – induced DNA demethylation and validation by pyrosequencing and quantitative real time PCR

The cell line UT-SCC-29 was seeded on the 6–well plate and cultured to reach 10–20% confluence under the same conditions as described in section 1.1. The cell line was incubated with 0.1 μM and 0.3 μM concentrations of 5-Aza-2′-deoxycytidine (Decitabine; DAC) in the culture medium. The medium, supplemented with freshly prepared DAC solution was replaced every 24 hours. For the control culture the same conditions, except the DAC solution replaced by equal volume of 50% acetic acid (DAC solvent) were used. Additionally, the “mock” control, i.e. the UT-SCC-29 cell line cultured only with DMEM medium, without any additional treatment was included. After reaching 80% of confluence, cells were harvested and DNA and RNA were isolated (as described above) and the effect of Decitabine application was assessed by bisulfite pyrosequencing and real-time qPCR.

DNA was isolated from the UT-SCC-29 cell line culture, treated either with DAC or acetic acid or untreated (“mock”) and bisulfite pyrosequencing of *FAM107A* promoter was performed using the same conditions as earlier in this study.

Likewise, RNA isolated from the respective cultures was reverse transcribed using Maxima First Strand cDNA Synthesis Kit (Thermo Fisher Scientific), according to manufacturer’s protocol. Primers for RT-qPCR were designed with the application of Beacon Designer^TM^ 7.5 software (PRIMER Biosoft International) in a way to include the intron localized between two coding exons of *FAM107A*. The primer sequences were verified with primer BLAST database (http://blast.ncbi.nlm.nih.gov/Blast.cgi) to confirm their specificity. As the reference, *GAPDH* gene was used. Primer sequences are presented in Table 3. The RT-qPCR reaction was performed with the use of HOT FIREPol® EvaGreen® qPCR Mix Plus (no ROX) (Solis BioDyne, Estonia), according to manufacturer’s protocol. No template control (NTC) was included to indicate lack of PCR contaminations. RT-qPCR was performed with the use of CFX96 Real-Time System (BIO-RAD) and the reaction conditions are described in Supplementary Table S3. The melting curve was generated to verify the product specificity and the PCR efficiency was appointed to estimate the relative expression value of *FAM107A*, as described previously^34^. All calculations were performed using Gene Expression MacroTM 1.10 software.

### Analysis of protein expression by immunohistochemistry

The samples designated for FAM107A protein analysis by IHC consisted of 15 primary LSCC and 5 non-tumor controls. All samples were processed in the Department of Clinical Pathology, Collegium Medicum, Nicolaus Copernicus University in Bydgoszcz, Poland and the previously described procedures of immunohistochemistry staining were applied^35, 36^. The primary rabbit polyclonal anti-FAM107A antibody (Thermo Fisher Scientific; cat. No: PA5-50409) was applied to estimate FAM107A protein expression.

The protocol was standardized using a series of positive and negative control reactions. The positive control reaction was performed on a model tissue selected according to The Human Protein Atlas (http://www.proteinatlas.org) and the antibody datasheet^37^. Therefore, consecutive 3 µm tissue sections of non-tumor fallopian tube sample were cut and used subsequently for IHC staining. The presence of the analyzed antigen was evaluated in glandular cells of fallopian tube. The nuclear/cytoplasmic/membranous expression was considered as positive FAM107A protein expression, according to reference sources. All negative control reactions were performed on additionally analyzed tissue sections, by substituting the primary antibody with a solution of 1% BSA (bovine serum albumin) diluted in PBS (phosphate buffered saline).

The paraffin blocks were cut on the manual rotary microtome (AccuCut, Sakura, Torrance, USA). 3 µm paraffin sections were prepared and mounted onto the extra adhesive slides (SuperFrostPlus, MenzelGlasser, Braunschweig, Germany). Deparaffinization, rehydratation and antigen retrieval were performed by heating sections in Epitope Retrieval Solution high-pH at 95–98 °C for 20 min. (Dako, Agilent Technologies, USA) in PT-Link (Dako). Subsequently, endogenous peroxidase activity was blocked with the use of 3% H_2_O_2_ solution for 15 minutes in room temperature (RT) and the non-specific binding was blocked using 5% solution of BSA for 15 minutes in RT. Incubation with the primary rabbit polyclonal anti-FAM107A antibody (dilution 1:200) was performed overnight at 4 °C. The antibody complex was detected using EnVisionFlex Anti-Mouse/Rabbit HRP-Labeled Polymer (Dako, Agilent Technologies) and localized using 3–3′diaminobenzidine (DAB) as chromogen. Finally, tissue sections were counterstained in hematoxylin, and subsequently dehydrated, cleared in series of xylenes, and coverslipped using mounting medium (Dako, Agilent Technologies).

The evaluation of protein expression was performed at 20x original objective magnification, in the light microscope ECLIPSE E400 (Nikon Instruments Europe, Amsterdam, Netherlands). For evaluation of FAM107A expression, immunohistochemical reactions were scored on a two-point qualitative scale:

0 – absence of FAM107A staining;

1 – presence of nuclear/cytoplasmic/membranous staining of FAM107A protein.

### Statistical analysis

The statistical calculations were performed using non-parametric Mann-Whitney test (GraphPad Prism 7 demo version) and chi-square test (http://quantpsy.org)^38^. Significance level was defined as *p* value less than 0.05 (*p* < 0.05). The box plot charts (Fig. 1) were prepared with the use of GraphPad Prism 7 demo version.

## Electronic supplementary material

Supplementary Materials

## References

[1] Torre LA (2015). Global cancer statistics, 2012. CA Cancer J Clin.

[2] Bonner JA (2006). Radiotherapy plus cetuximab for squamous-cell carcinoma of the head and neck. N Engl J Med.

[3] Leemans CR, Braakhuis BJ, Brakenhoff RH (2011). The molecular biology of head and neck cancer. Nat Rev Cancer.

[4] Califano J (1996). Genetic progression model for head and neck cancer: implications for field cancerization. Cancer Res.

[5] Agrawal N (2011). Exome sequencing of head and neck squamous cell carcinoma reveals inactivating mutations in NOTCH1. Science.

[6] Nadal A, Cardesa A (2003). Molecular biology of laryngeal squamous cell carcinoma. Virchows Arch.

[7] Jarmuz-Szymczak M (2013). Heterogeneity of 11q13 region rearrangements in laryngeal squamous cell carcinoma analyzed by microarray platforms and fluorescence *in situ* hybridization. Mol Biol Rep.

[8] Hunt JL (2014). Molecular diagnostic alterations in squamous cell carcinoma of the head and neck and potential diagnostic applications. Eur Arch Otorhinolaryngol.

[9] Guerrero-Preston R (2014). Key tumor suppressor genes inactivated by “greater promoter” methylation and somatic mutations in head and neck cancer. Epigenetics.

[10] The Cancer Genome Atlas Network. Comprehensive genomic characterization of head and neck squamous cell carcinomas. *Nature* **517**, 576-582, doi:10.1038/nature14129 (2015). 10.1038/nature14129 PMC4311405 25631445

[11] Giefing M (2016). Moving towards personalised therapy in head and neck squamous cell carcinoma through analysis of next generation sequencing data. Eur J Cancer.

[12] Giefing M (2011). High resolution ArrayCGH and expression profiling identifies PTPRD and PCDH17/PCH68 as tumor suppressor gene candidates in laryngeal squamous cell carcinoma. Genes Chromosomes Cancer.

[13] Giefing M (2008). Characterization of homozygous deletions in laryngeal squamous cell carcinoma cell lines. Cancer Genet Cytogenet.

[14] Vogelstein B (2013). Cancer genome landscapes. Science.

[15] Knudson AG (1971). Mutation and cancer: statistical study of retinoblastoma. Proc Natl Acad Sci USA.

[16] Hogg RP (2002). Frequent 3p allele loss and epigenetic inactivation of the RASSF1A tumour suppressor gene from region 3p21.3 in head and neck squamous cell carcinoma. Eur J Cancer.

[17] Tai SK (2004). Loss of Fhit expression in head and neck squamous cell carcinoma and its potential clinical implication. Clin Cancer Res.

[18] Lee DJ (2010). Multiple tumor-suppressor genes on chromosome 3p contribute to head and neck squamous cell carcinoma tumorigenesis. Cancer Biol Ther.

[19] Wang L (2000). Loss of expression of the DRR 1 gene at chromosomal segment 3p21.1 in renal cell carcinoma. Genes Chromosomes Cancer.

[20] van den Boom J, Wolter M, Blaschke B, Knobbe CB, Reifenberger G (2006). Identification of novel genes associated with astrocytoma progression using suppression subtractive hybridization and real-time reverse transcription-polymerase chain reaction. Int J Cancer.

[21] Awakura Y, Nakamura E, Ito N, Kamoto T, Ogawa O (2008). Methylation-associated silencing of TU3A in human cancers. Int J Oncol.

[22] Yamato T, Orikasa K, Fukushige S, Orikasa S, Horii A (1999). Isolation and characterization of the novel gene, TU3A, in a commonly deleted region on 3p14.3–p14.2 in renal cell carcinoma. Cytogenet. Cell Genet.

[23] Szukala K (2006). Does loss of heterozygosity in critical genome regions predict a local relapse in patients after laryngectomy?. Mutat Res.

[24] Cerami E (2012). The cBio cancer genomics portal: an open platform for exploring multidimensional cancer genomics data. Cancer Discov.

[25] Gao J (2013). Integrative analysis of complex cancer genomics and clinical profiles using the cBioPortal. Sci Signal.

[26] Udali S (2015). DNA methylation and gene expression profiles show novel regulatory pathways in hepatocellular carcinoma. Clin Epigenetics.

[27] Pastuszak-Lewandoska D (2015). Decreased FAM107A Expression in Patients with Non-small Cell Lung Cancer. Adv Exp Med Biol.

[28] Nervi C, De Marinis E, Codacci-Pisanelli G (2015). Epigenetic treatment of solid tumours: a review of clinical trials. Clin Epigenetics.

[29] Liu Q (2009). Induction of tumor inhibition and apoptosis by a candidate tumor suppressor gene DRR1 on 3p21.1. Oncol Rep.

[30] Jarmuz M, Golusinski W, Grenman R, Szyfter K (2002). Analysis of chromosome aberrations in cell lines derived from laryngeal cancer in relation to tumor progression. Eur Arch Otorhinolaryngol.

[31] Jarvinen AK (2006). Identification of target genes in laryngeal squamous cell carcinoma by high-resolution copy number and gene expression microarray analyses. Oncogene.

[32] Chomczynski, P. A reagent for the single-step simultaneous isolation of RNA, DNA and proteins from cell and tissue samples. *Biotechniques* **15**, 532–534, 536–537 (1993). 7692896

[33] Szaumkessel M (2011). Pyrosequencing-based DNA methylation profiling of Fanconi anemia/BRCA pathway genes in laryngeal squamous cell carcinoma. Int J Oncol.

[34] Kostrzewska-Poczekaj M (2010). Recurrent amplification in the 22q11 region in laryngeal squamous cell carcinoma results in overexpression of the CRKL but not the MAPK1 oncogene. Cancer Biomark.

[35] Bodnar M, Szylberg L, Kazmierczak W, Marszalek A (2015). Tumor progression driven by pathways activating matrix metalloproteinases and their inhibitors. J Oral Pathol Med.

[36] Bodnar M (2016). Proteomic profiling identifies the inorganic pyrophosphatase (PPA1) protein as a potential biomarker of metastasis in laryngeal squamous cell carcinoma. Amino Acids.

[37] Uhlen, M. *et al*. Towards a knowledge-based Human Protein Atlas. *Nat Biotechnol* **28**, 1248–1250, doi:nbt1210-1248 (2010). 10.1038/nbt1210-1248 21139605

[38] Preacher, K. J. Calculation for the chi-square test: An interactive calculation tool for chi-square tests of goodness of fit and independence [Computer software]. http://quantpsy.org (2001).

