# Supplementary material for: Combined deletion and DNA methylation result in silencing of FAM107A gene in laryngeal tumors
Source: Sci Rep. 2017 Jul 14;7:5386. doi: 10.1038/s41598-017-05857-1 (PMC5511162; doi:10.1038/s41598-017-05857-1)

Combined deletion and DNA methylation result in silencing of *FAM107A* gene in laryngeal tumors

Katarzyna Kiwerska[1,2*], Marcin Szaumkessel[1], Julia Paczkowska[1], Magdalena Bodnar[3,4], Ewa Byzia[1], Ewelina Kowal[1], Magdalena Kostrzewska-Poczekaj[1], Joanna Janiszewska[1], Kinga Bednarek[1], Małgorzata Jarmuż-Szymczak[1,5], Ewelina Kalinowicz[4], Małgorzata Wierzbicka[4], Reidar Grenman[6], Krzysztof Szyfter[1,7], Andrzej Marszałek[3,8] and Maciej Giefing[1,4]

1. Institute of Human Genetics, Polish Academy of Sciences, Strzeszyńska 32, 60-479 Poznań, Poland
2. Department of Tumor Pathology, Greater Poland Cancer Center, Garbary 15, 61-866 Poznań, Poland
3. Department of Clinical Pathomorphology, Collegium Medicum in Bydgoszcz, Nicolaus Copernicus University in Toruń, Curie-Sklodowskiej 9, 85-094 Bydgoszcz, Poland
4. Department of Otolaryngology and Laryngeal Oncology, Poznan University of Medical Sciences, Przybyszewskiego 49, 60-355 Poznań, Poland
5. Department of Hematology and Bone Marrow Transplantation, Poznan University of Medical Sciences, Szamarzewskiego 82/84, 60-569 Poznań, Poland
6. Department of Otorhinolaryngology, Head and Neck Surgery and Department of Medical Biochemistry, Turku University Central Hospital and Turku University, PO Box 52 FI-20521 Turku, Finland
7. Department of Audiology and Phoniatrics, Poznan University of Medical Sciences, Przybyszewskiego 49, 60-355 Poznań, Poland
8. Oncologic Pathology and Prophylaxis Poznan University of Medical Sciences & Greater Poland Cancer Center, Garbary 15, 61-866 Poznań, Poland

*Corresponding author: katarzyna.kiwerska@igcz.poznan.pl
Phone +48 61 65 79 214
Fax +48 61 82 33 235

Supplementary Table S1. Cell line characteristics.

Cell line	Sex	Age (years)	Primary tumor	TNM	Grade
UT-SCC-4	F	43	hypophar supraglottic larynx	$T_3N_0M_0$	G2
UT-SCC-6A	F	51	supraglottic larynx	$T_2N_1M_0$	G1
UT-SCC-6B	F	51	supraglottic larynx	$T_2N_1M_0$	G1
UT-SCC-11	M	58	glottic larynx	$T_1N_0M_0$	G2
UT-SCC-19B	M	44	glottic larynx	$T_4N_0M_0$	G2
UT-SCC-22	M	79	glottic larynx	$T_1N_0M_0$	G2
UT-SCC-23	M	66	scc transglottica	$T_3N_0M_0$	G1
UT-SCC-29	M	82	glottic larynx	$T_2N_0M_0$	G1
UT-SCC-34	M	63	supraglottic larynx	$T_4N_0M_0$	G1
UT-SCC-35	M	50	glottic larynx	$T_2N_0M_0$	G2
UT-SCC-38	M	66	glottic larynx	$T_2N_0M_0$	G2
UT-SCC-42B	M	43	supraglottic larynx	$T_4N_3M_0$	G3
UT-SCC-57	M	76	glottic larynx	$T_2N_0M_0$	G1-G2
UT-SCC-106A	M	59	SCC plicae vocalis	$T_{1A}N_0M_0$	G2
UT-SCC-107	M	46	SCC laryngis supraglottidis	$T_4N_{2C}M_0$	G2
UT-SCC-108	M	68	SCC laryngis supraglottidis	$T_2N_0M_0$	G3
UT-SCC-116	M	60	SCC laryngis supraglottidis	$T_4N_1M_0$	G2

Supplementary Table S2. The clinical data of patients and primary tumors.

No	Sex	Age	TNM	G
MK2	M	59	T3N2M0	2
MK3	M	64	T3N2M0	2
MK15	M	57	T3N1M0	1
MK19	M	65	T4N3M1	1
MK20	M	61	T3N3M0	2
MK22	K	60	pT2N1M0	2
MK31	K	61	T4N3M0	3
MK32	M	61	T3N0M0	2
MK34	M	61	T4N1M0	2
MK35	M	72	T3N0M0	1
MK36	M	53	T4N1M0	2
MK41	M	64	T3N0M0	2
MK42	M	69	T2N2cM0	2
MK44	M	54	T4N0M0	2
MK47	M	73	T4N0M0	3
MK48	M	62	T4N0M0	2
MK56	M	71	T4N0M0	1
MK59	M	59	T3N2M0	2
MK60	M	71	T4N3M0	2
MK62	M	66	T4N2M0	2
MK63	M	66	T4N2M0	3

Supplementary Table S3. The conditions of performed experiments.

PR in the RT-qPCR section indicates “plate read” i.e. fluorescence data collection

Study	Step		Temp [°C]	Time
RT-PCR	Initial denaturation		95	3 min.
	Denaturation	35 cycles	95	30 sec.
	Annealing		55	20 sec.
	Elongation		72	20 sec.
	Final elongation		72	7 min.
	Hold		4	∞
pre SEQ PCR	Initial denaturation		95	5 min.
	Denaturation	35 cycles	95	30 sec.
	Annealing		65	30 sec.
	Elongation		72	20 sec.
	Final elongation		72	5 min.
	Hold		4	∞
PYROSEQ PCR	Initial heat activation		95	15 min.
	Denaturation	45 cycles	94	30 sec.
	Annealing		55	30 sec.
	Extension		72	30 sec.
	Final extension		72	10 min.
RT-qPCR	Initial polymerase activation		95	15 min.
	Denaturation	40 cycles	95	15 sec.
	Annealing		52	20 sec.
	Elongation + PR		72	20 sec.
	Melt curve: 50-95°C in 0.5°C/10sec. increments + PR			

Supplementary Figure S1. The graphs showing deletions of 3p arm in laryngeal cancer cell lines. UT-SCC-11, -22 and -34 were analyzed with the use of Agilent Human Genome CGH 44K Microarray and the remaining cell lines – with the application of Agilent Human Genome CGH 244A Microarray.

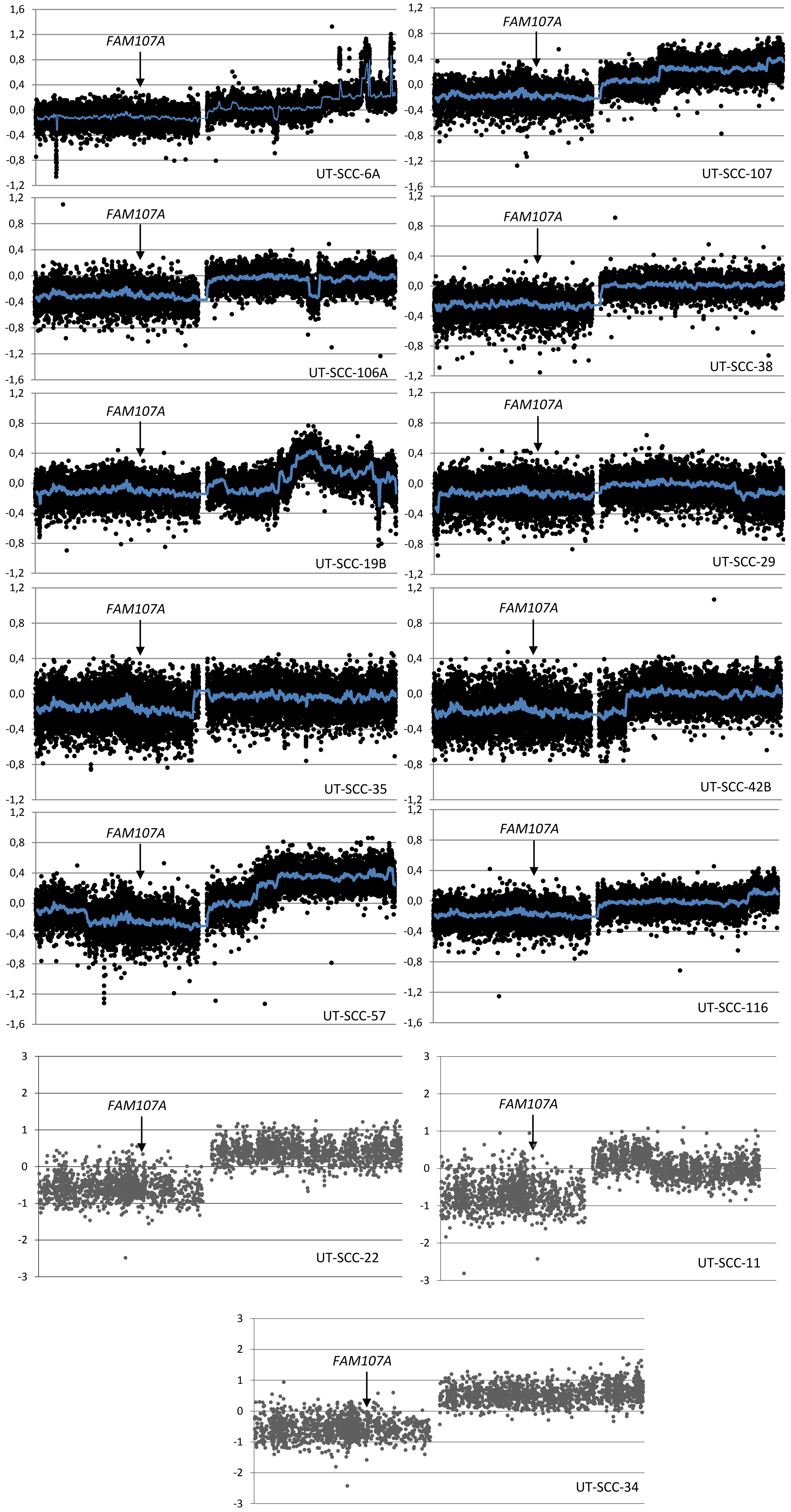

Supplementary Figure S2. Electropherograms of SNPs detected in *FAM107A* gene.

Frames indicate the altered nucleotides.

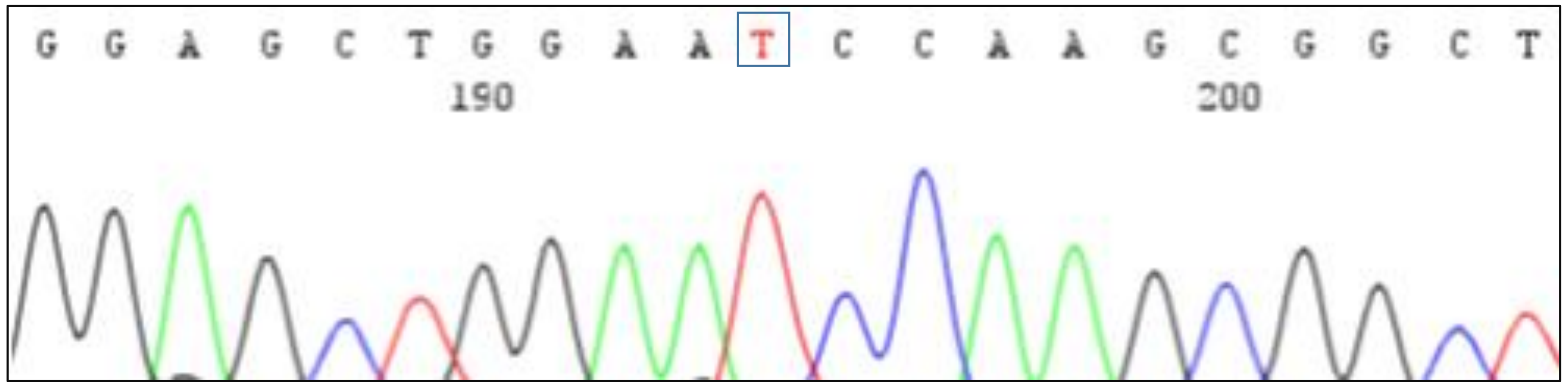

A. rs1043942 c.265G>T

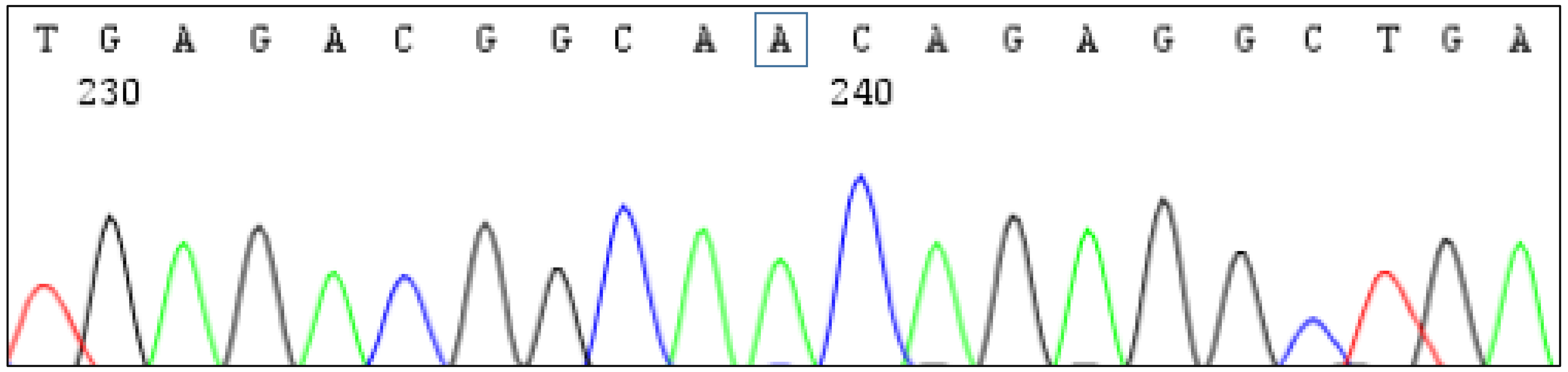

B. rs1139701 c.312G>A

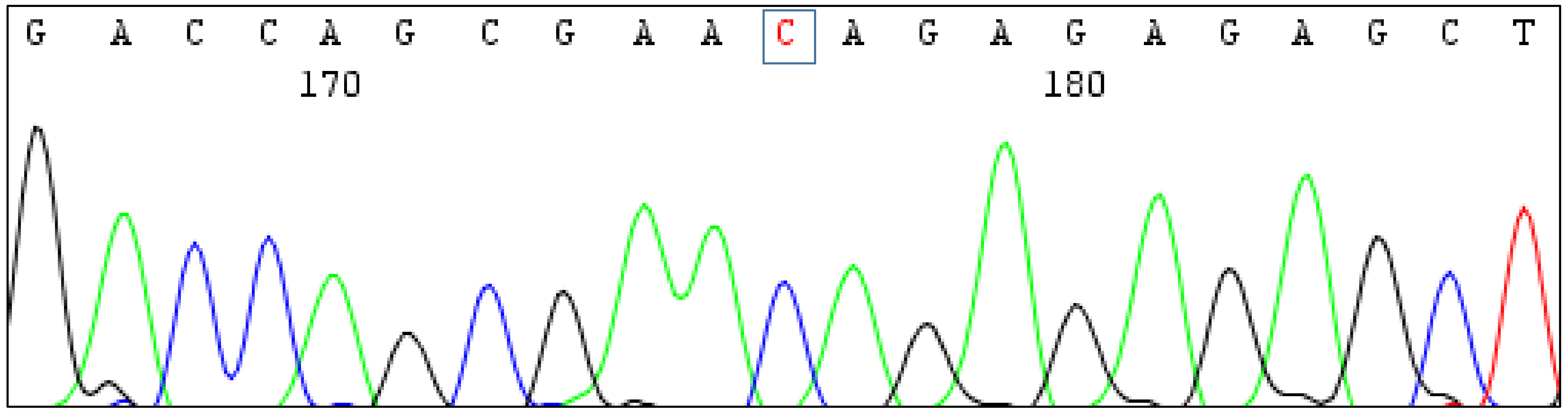

C. rs11539086 c.421G>C

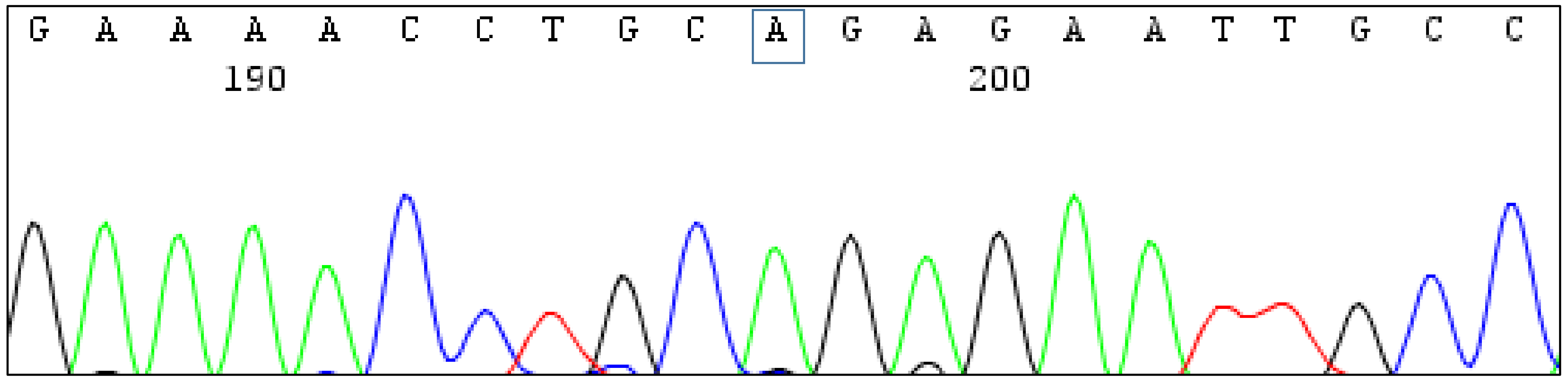

D. rs141609445 c.395G>A

Supplementary Figure S3. The serial dilutions of methylated DNA (sensitivity control).

The star indicates that above the cut off value (41%) DNA is hypermethylated at 70%.

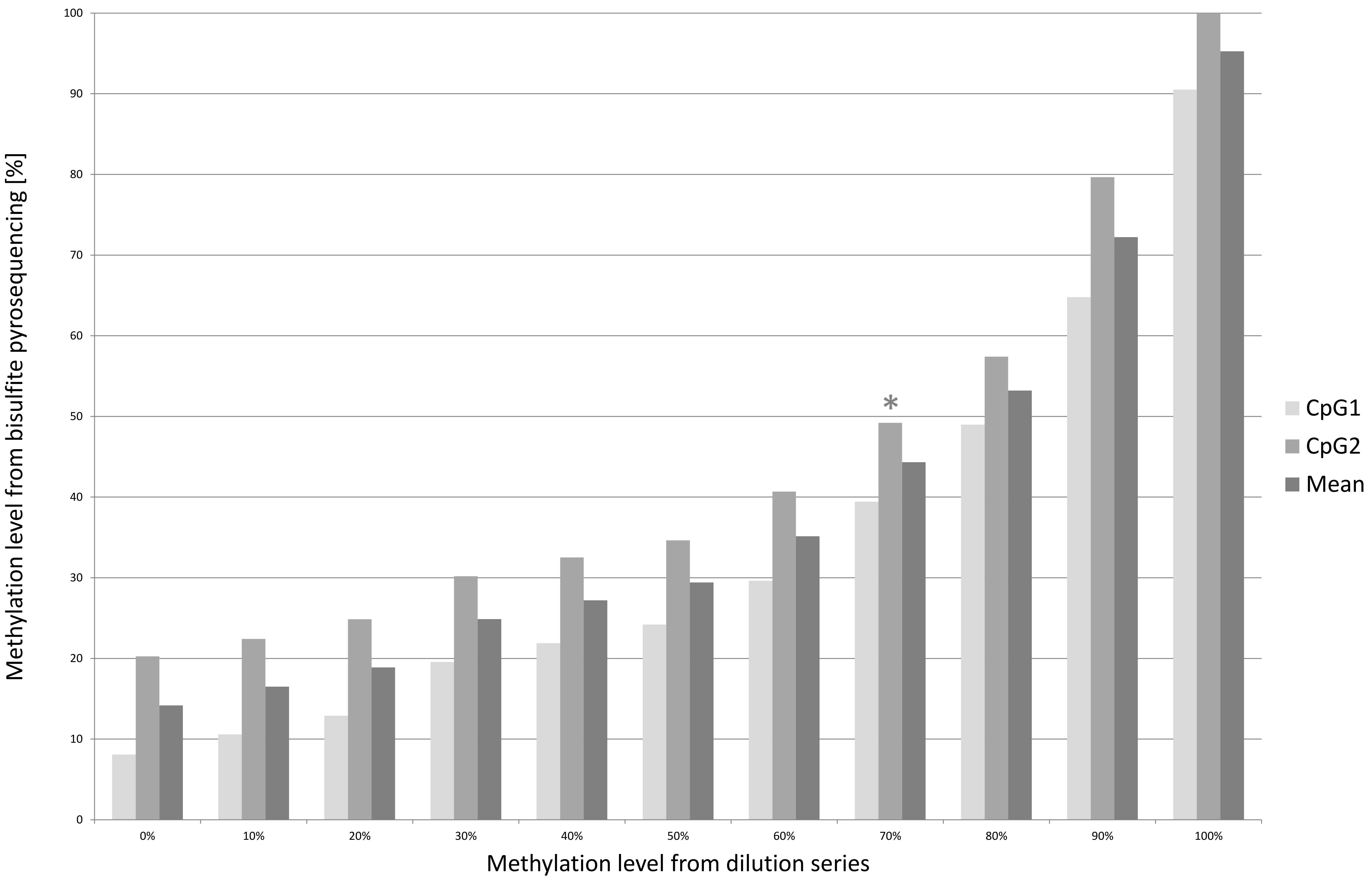

Supplement: Supplementary file 1 — Supplementary Materials [file 41598_2017_5857_MOESM1_ESM.pdf]
